# Consultations in primary care for symptoms attributed to electromagnetic fields – a survey among general practitioners

**DOI:** 10.1186/1471-2458-6-267

**Published:** 2006-10-30

**Authors:** Anke Huss, Martin Röösli

**Affiliations:** 1 Department of Social and Preventive Medicine, University of Berne, Finkenhubelweg 11, 3012 Berne, Switzerland

## Abstract

**Background:**

Five percent of the Swiss population attribute symptoms to electromagnetic fields (EMF). General practitioners (GPs) might play a key role in recognising an emerging health risk, since they are the first to observe and follow up persons who attribute symptoms to EMF. It is unclear to what extent EMFs have become an issue in general practice and which experiences GPs report from the consultations.

**Methods:**

We conducted telephone interviews in a random sample of GPs in Switzerland in order to assess the frequency of consultations in primary care due to EMF and the GPs' experience with these patients.

**Results:**

342 general practitioners were interviewed, corresponding to a response rate of 28.2%. 69% of the GPs reported at least one consultation due to EMF, but GPs with a certificate in complementary medicine were much more likely to report EMF consultations. The median of EMF consultation numbers within one year was three. An overview of the most recent EMF-related consultation per GP yielded sleep disorders, headaches and fatigue as the most often reported symptoms and mobile phone base stations, power lines and the own use of mobile phones as the main EMF sources suspected to be associated to symptoms. GPs judged the association between EMF and the symptoms to be plausible in 54% of the cases. There was no combination of symptoms and EMF sources that was remarkably and consistently judged to be a plausible cause of the symptoms.

**Conclusion:**

In our survey, GPs often judged the association between the health problems and the suspected exposure to be plausible. This plausibility assessment seems to be based on grounds of preventive positions in a situation of scientific uncertainty. More research effort is needed to obtain more insight on a potential association between long term EMF exposure and unspecific symptoms.

## Background

Everyone is exposed to a complex mixture of electric and magnetic fields at many different frequencies. In the scientific literature and in the media, increasing attention is paid to the potential adverse effects of electromagnetic fields (EMF) on human health, especially since the introduction of modern telecommunication technologies. Regarding radiofrequency EMF, such as those from base stations or mobile phones, the Stewart Report concluded in 2000 that "the balance of evidence to date suggests that exposures to RF radiation below guidelines issued by the National Radiological Protection Board (NRPB) and the International Commission on Non-Ionizing Radiation Protection (ICNIRP) guidelines do not cause adverse health effects to the general population" [1]. In 2004, a review of 26 reports on mobile phones and health by the NRPB concluded that exposure towards radiofrequency EMFs causing adverse health effects remained unproven. There was, however, scientific evidence suggesting subtle biological effects [2]. The World Health Organization summarised in a report on low-frequency exposures, such as from household or railway power supply, that "many health outcomes ranging from reproductive defects to cardiovascular and neurodegenerative diseases have been examined, but the most consistent evidence to date concerns childhood leukaemia" [3]. The International Agency for Research on Cancer classified low-frequency magnetic fields as possibly carcinogenic to humans based on epidemiological studies of childhood leukaemia [4].

So far, it is unclear to what extent EMFs have become an issue in general practice and which experiences GPs report from these consultations. In 2005, an estimate based on a representative survey yielded that more than half of the Swiss population perceived EMFs as potentially harmful and 5% attributed symptoms to EMF [5]. Of these affected persons, 13% reported to having consulted a general practitioner (GP). GPs might play a key role in recognising an emerging health risk, since they are the first to observe and follow up persons who report health problems.

We conducted computer assisted telephone interviews (CATI) on a random sample of GPs in Switzerland in order to assess the frequency of consultations in primary care due to EMF, their experience with these patients as well as their perception of risk related to EMFs.

## Methods

General practitioners in the French and German speaking parts of Switzerland working in primary care practices were eligible for the survey. A random sample was drawn from the address-database of the Swiss Medical Association, stratified by language area.

The survey was announced by a letter beforehand. We interviewed GPs by means of CATI. The time frame was between 15–23 minutes per interview, depending on whether GPs reported having had any consultations about EMF or not. The interviews took place in May and June 2005.

In order to obtain a representative overview over occurring cases, GPs were asked about the most recent EMF case: They were inquired about reported symptoms and suspected EMF sources, an estimation of the plausibility of an association between EMF source and health complaint(s) by the GP and the given advice. They were also asked about the frequency of such consultations. Another part of the questionnaire dealt with the GPs' perception of potential health risks from EMF and their self-reported level and/or need of information. We also asked whether GPs thought that it would be necessary to implement a national or regional interdisciplinary environmental medicine counselling centre. The questions were openly asked with a prepared list for the interviewers in order to avoid suggestion bias.

GPs with a certificate in traditional Chinese medicine or acupuncture (Association of Swiss Practitioners' Societies for Acupuncture and Chinese Medicine, "ASA"), homeopathy (Swiss Association of Homeopathic Physicians, "SVHA"), neural therapy (Swiss Medical Association for Neural Therapy, "SANTH") or anthroposopical medicine (Anthroposophic Medical Association of Switzerland, "VAOAS") were grouped as "complementary-medicine GPs".

We calculated percentages of subgroups of GPs who reported at least one EMF consultation or who believed that EMF, as occurring under everyday conditions, could cause symptoms; 95% confidence intervals for proportions were estimated with the Wilsons 'score' method [6]. We then evaluated which factors predicted whether GPs reported EMF consultations or that GPs believed that EMF can cause symptoms using multiple logistic regression. We used the same procedure to compare GPs "plausible" case evaluations to "implausible" evaluations. We included in all our logistic regression models sex and age of the GP, whether the majority of the patients of each practice came from "urban or agglomeration", "rural", or "equally urban or rural" areas, self rated information level with regard to health effects from EMF (rather bad, middle, rather good) as well as complementary-medicine certificate (yes versus no). Analyses were performed in STATA 9 (StataCorp, College Station, Texas, USA).

## Results

Of approximately 7200 GPs in Switzerland, we contacted 1328 (18%). Of these, 375 agreed to the interview, corresponding to a response rate of 28.2%. Thirty-three persons did not fulfil eligibility criteria (not currently practicing as GP) which resulted in 342 completed interviews. The mean age was 52 years (SD 7.7 years, range 36 – 75) and 20% were female (Table 1). Fifty-eight (17%) of the GPs had at least one certificate in complementary-medicine. These were 8% with a certificate in traditional Chinese medicine, 7% in homeopathy, 5% in anthroposopical medicine and 1.5% in neural therapy.

EMF as a reason for at least one consultation in the past were reported by 69% of all GPs (Table 2). Complementary-medicine GPs were much more likely to report the occurrence of at least one EMF consultation (Table 3) as were those GPs who reported a higher self-rated level of information with regard to potential health effects from EMF.

The majority of their consultations to do with EMFs had occurred within the last year and 19% of the GPs reported to have had 10 or more such consultations over the course of the last year. Complementary-medicine GPs reported a higher frequency of EMF-related consultations with a median of 10 (interquartile range 3 – 35) compared to non-complementary-medicine GPs with 3 (interquartile range 1 – 5.5). Of 171 GPs who had had their practice for over a decade, half (50%) reported an increase of the consultation numbers over that time, 43% reported no increase and 7% didn't know.

**Figure 1:**
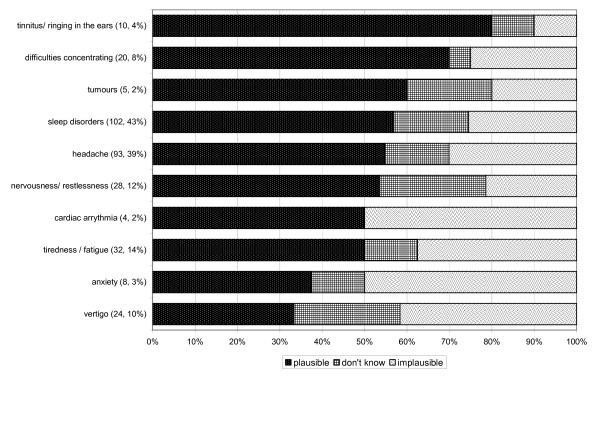
Plausibility rating of association between symptoms and their suspected source, by symptom (absolute number as well as percentage of cases who reported the respective symptom in brackets).

**Figure 2:**
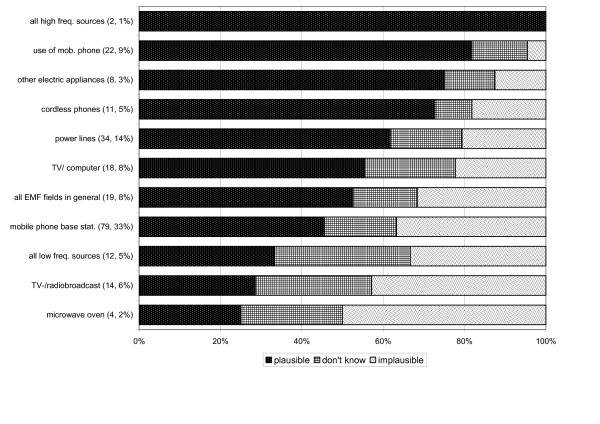
Plausibility rating of association between suspected source and symptoms, by source (absolute number as well as percentage of cases who suspected this EMF source in brackets).

When asked about details of their most recent EMF consultations, symptoms of patients were usually non-specific. Symptoms that were reported more than once included sleep disorders (43% of cases), headaches (39%), fatigue (14%), and a range of other health complaints, such as nervousness, vertigo, difficulties concentrating, tinnitus, anxiety, tumours and cardiac arrhythmias (Figure 1). The most often listed EMF sources suspected to be associated with the symptoms were mobile phone base stations (33% of cases), power lines (14%) and the use of mobile phones (9%), but other reported specific sources included e.g. TV or computers, TV- or radio broadcasting stations, cordless telephones or microwave ovens. More general suspected sources included "all EMF sources", "all communication technology EMF", "all EMF from household or train power supplies" (Figure 2). There were no clear patterns between the reported symptoms and their suspected sources.

In most of the cases (77%), the patients suggested their suspected association between an EMF source and the health complaint in the consultation but, in a minority of cases, only the GP, or both GP and patient (11% each) thought of EMF as a potential cause of the symptoms. In 1% of the cases the GPs could not remember who suggested the association. Depending on who reported the association, (GP, patient, both), the most often suspected EMF-sources varied, but not the listed health complaints. For example, if the patient suspected EMF as cause of the health problem, mobile phone base stations were most often suspected as being the cause (37%), but not the own use of mobile phones (8%). If the association was brought up by the GP, mobile phone use was most often suspected (24% of cases) whereas mobile phone base stations were only suspected in 4%. The cases where both GP and patient brought up the association were quite similar to those where the patient alone had brought up EMF as a suspected cause (35% mobile phone base stations, 8% mobile phone use). Power lines as the suspected source were equally often subject of the consultation, independently on who thought about this source first (patients, GPs or both: 14, 16 and 15%, respectively).

In more than half (54%) of the cases, the GPs judged the association between EMF and symptoms to be "plausible" and in 29% to be "implausible". Seventeen percent didn't know. Comparison of "plausible" versus "implausible" ratings showed that age or sex of the GP had no effect on these ratings, but other variables had. Complementary-medicine GPs were more likely to evaluate the association as "plausible", compared to the rest of the GP group (OR: 16.8, 95% Confidence Interval 3.8 – 74.5). There were some symptoms that were more often thought to be plausible than others, especially tinnitus (80% plausible ratings), concentration difficulties (70%) and tumours (60%). In contrast, cardiac arrhythmias, anxiety or vertigo were most frequently though to be implausibly connected to EMF sources (50%, 50% and 42%, respectively) (Figure 1). Of the EMF sources, the own use of mobile phones, electric equipment and cordless telephones – sources in close proximity to the body – were more often judged to be a plausible cause of the symptoms (82%, 75% and 73%, respectively), compared to other suspected sources. Microwave ovens, TV or radiobroadcast stations as well as mobile phone base stations were most often thought to be implausibly connected to the symptoms (50%, 43% and 37%, respectively) (Figure 2). There was no combination of symptoms and EMF sources that was remarkably and consistently judged to be plausible.

The given counsel could be grouped into three main categories: exposure-focussed advice, treatment-focussed advice (e.g. medication or psychosomatic intervention) or no advice. Most often, patients were given exposure-focussed advice with 40% of the consultations where the patients were advised to get rid of the EMF source, e.g. by switching the source off, move house, avoiding the EMF when possible, etc. GPs reported that in 43% of the patients, an improvement in the state of health had occurred. This was independent of any counselling or therapies administered by them.

Asked in a more general sense, the majority of the GPs (61%) believed that exposure to EMFs as they occur under everyday conditions, can cause symptoms, 27% disagreed and 12% had no opinion. Female GPs were more likely to think that EMFs can cause symptoms, as well as complementary-medicine GPs (Table 4). 14% of the GPs had considered at least once whether EMF could have been the cause for own symptoms. Again, complementary-medicine GPs were more likely to have had considered EMF as potential cause of symptoms, compared to non-complementary GPs (OR 4.2, 95% C.I. 2.1 – 9.0).

## Discussion

EMF as a reason for at least one consultation in the past were reported by the majority of the GPs. An overview of the most recent EMF-related consultations in general practice yielded sleep disorders, headaches and fatigue as the most often reported symptoms and mobile phone base stations, power lines and the own use of mobile phones as the main EMF sources suspected to be associated with these symptoms. GPs judged the relation between EMF and the symptoms to be "plausible" in more than half of the cases.

Our response rate of 28.2% was low. A potential problem could be a selection bias in which concerned or affected GPs were more likely than others to participate. We found an indirect indication for this, because complementary-medicine GP were somewhat overrepresented in our GP sample compared to the data base for all GPs. Complementary-medicine GPs were much more likely to report consultations because of electromagnetic fields, to believe that exposure to EMFs as they occur under everyday conditions can cause symptoms and to have considered whether EMF could have been the cause for own symptoms. Weighting the complementary-medicine GPs answers by their relative occurrence in the population of GPs as a whole reduced our estimates only slightly: 67% instead of 69% of GPs reported at least one EMF-consultation, 59% instead of 61% believed that EMF could cause symptoms and 12% instead of 14% had considered EMFs as potential cause for symptoms they had experienced themselves.

In Austria, a written survey on EMF-consultations in general practice yielded very similar results to our survey, although the response rate in the Austrian survey was considerably higher at 49% [7]. Sixty-eight percent of the GPs reported to have "sometimes" or "frequently" EMF consultations. With respect to risk perception, the GPs were even more concerned about potential health effects from EMF exposure than our Swiss sample.

In a survey based on the Swiss Sentinel system, environmental medicine consultations were assessed over the course of the year 2002 [8]. In this survey, 30% of the GPs reported at least one environmental medicine consultation within that year, and 2–12% of these consultations were due to EMFs. This is much lower than the percentage found in our survey, where 58% of all GPs reported at least one EMF consultation within the last year. One of the reasons for this discrepancy could be a real increase in frequency of these consultations since 2002. In our survey, GPs with more than 10 years experience in their practice reported to have observed an increase in EMF-related consultations. Another reason could be that our telephone interview was announced by a letter, which might have led to remembering a case that could otherwise have gone forgotten. This could occasionally have happened in the Sentinella survey where GPs had to fill in a questionnaire once a week. In addition, in the Sentinella survey, GPs were asked about a range of environmental exposures. Exposure towards mobile phones, however, might be considered to be live-style related, rather than to be an environmental exposure. This would also result in reporting fewer cases in comparison to our survey. As in the Sentinella survey [8], the complementary medicine GPs in our survey were less likely than the other GPs to reject an association between environmental exposures and their putative adverse health effects. This may account for the larger number of reported cases and higher attendance of concerned persons in such practices.

Overall, GPs often believed that an association between symptoms and EMF was plausible and frequently advised affected patients to avoid or reduce exposure. Interestingly, a range of international expert panels so far have concluded that the possibility of EMF exposure causing adverse health effects remains unproven [9-17]. This might be considered as a discrepancy. However, one has to take into account that there are two differing concepts behind this apparent discrepancy: the population-based causality evaluation of expert panels, and the individual-based plausibility assessment by the GPs. Single case evaluation is unlikely to provide sufficient evidence to demonstrate a causal relation between exposures and health outcomes, even in those situations where exposures have been shown to be related to health outcomes in epidemiological studies. We took account of these differing concepts by asking for "plausibility" rather than "causality" in our survey. This implicates, however, that GPs might just be prudent in not excluding EMF from a list of potentially possible exposures associated with the health complaints in a situation where causality cannot be addressed directly. Thus, the reported plausibility assessment from the GPs in our survey may be based on grounds of preventive positions in a situation of scientific uncertainty: There are only very few studies that evaluated unspecific symptoms in association with EMF exposure on a long term basis.

There was agreement between scientific expert panels and the interviewed GPs with respect to plausibility ratings according to localisation of EMF sources. The NRPB (2004)[2] reviewed 26 reports from expert panels on EMF and health and summarised that " [...] very low level exposures, typical of base stations, are extremely unlikely to cause any effects on biophysical grounds, whereas localised exposures, typical of those from mobile phones, may induce effects [...]." In our survey, EMF emitted close to the body (e.g. mobile phones, cordless phones and electrical appliances) were more often described as "plausible" causes of the health complaints, compared to sources further away (e.g. mobile phone base stations, TV or radio broadcasting stations). Apart from that, there are no obvious patterns that can be extracted from this survey, which would link specific EMF sources to specific health complaints and thus call for a new direction of investigation that is not yet pursued by researchers. However, a pattern would have to be fairly source and symptom specific so that we would be able to pick it up in a survey like ours.

Most of the GPs reported little experience with EMF consultations in general practice: 71% of all GPs reported to have had fewer than five EMF consultations in the previous year. Of the GPs with few or no EMF consultations in the previous year, the majority still estimated an association between health complaints and EMF sources to be plausible in 52% of the cases. This makes it unlikely that GPs develop their plausibility assessment based on frequent and repeated experience with EMF-related consultations.

There were some indications that GPs did not feel confident about counselling persons with symptoms attributed to EMF, e.g. 75% expressed to need more information on the topic for their work as a GP and less than half of the GPs reported to have a standard approach on how to deal with these patients. 53% would welcome the implementation of a national or regional interdisciplinary environmental medicine counselling centre.

## Conclusion

From the general practitioners' observations no obvious symptom-EMF source pattern could be extracted, which would link a specific EMF source to a specific health complaint and thus call for a new direction of investigation that is not yet pursued by researchers. However, the scientific uncertainty regarding long term effects of EMF on unspecific symptoms is mirrored in the GPs plausibility assessments: The majority of GPs believed that exposure to everyday EMF could cause symptoms, and the relation between EMF and the symptoms of their EMF-related consultations was judged to be "plausible" in more than half of the cases. Thus, we conclude that more research effort is needed to obtain more insight on a potential association between long term EMF exposure and unspecific symptoms.

## List of abbreviations used

CATI – computer assisted telephone interview

EMF – electromagnetic fields

GP – General Practitioner

ICNIRP – International Commission on Non-Ionizing Radiation Protection

NRPB – National Radiological Protection Board

## Competing interests

The author(s) declare that they have no competing interests.

## Authors' contributions

AH coordinated the study and drafted the manuscript and MR conceived of the study. Both authors designed the study, developed the questionnaire used in the survey, analysed and interpreted the data, revised the draft and read and approved the final manuscript.

## Pre-publication history

The pre-publication history for this paper can be accessed here:



## Figures and Tables

**Table 1:** Demographics of GPs

	**N, (%)**
***All GPs***	***342 (100)***

**Sex**	
Male	275 (80)
Female	67 (20)
**Age groups (years)**	
<35 – 44	64 (19)
45 – 54	126 (37)
55 – 64	138 (41)
≥ 65	12 (3)
**Majority of patients comes from rural vs. urban and agglomeration area**	
Majority from rural area	124 (36)
Majority from urban or agglomeration area	139 (41)
Equally urban or agglomeration/rural	78 (23)
**GPs self-reported level of information regarding health effects from EMF**	
Rather bad	74 (22)
Middle	173 (51)
Rather good	90 (26)
Don't know	5 (1)
**GPs with certificate in complementary medicine^a^**	
No certificate	284 (83)
With certificate	58 (17)

^a ^At least one of the following: traditional Chinese medicine/acupuncture, neural therapy, homeopathy or anthroposophical medicine

**Table 2:** number of EMF consultations.

**Most recent EMF consultation**	**N 342**	**%**
No EMF consultations reported	**105**	**30.7**
Reports at least one EMF-consultation	**237**	**69.3**

**Number of EMF consultations in the last year**		
0	32	9.4
1 – 4	97	28.4
5 – 9	36	10.5
10 – 49	48	14.0
50 – 99	10	2.9
≥ 100	6	1.8
Don't know	8	2.3

Number (%) of GPs reporting any EMF-consultation as well as the number of EMF-consultations within the last year.

**Table 3:** number and percentage of GPs who reported at least one EMF-consultation, stratified by GP-characteristics.

	***% (95% C.I.)***	***OR (95% C.I.)^a^***	***p^b^***
**all GPs**	***69 (64 – 74)***		

**Sex**			
Male	69.5 (63.8 – 74.6)	1 (reference)	
Female	68.7 (56.8 – 78.5)	0.79 (0.40 – 1.56)	0.50
**Age group (years)**			
<35 – 44	78.1 (66.6 – 86.5)	1 (reference)	
45 – 54	69.8 (61.3 – 77.2)	0.52 (0.24 – 1.1)	
55 – 64	65.2 (57.0 – 72.7)	0.48 (0.23 – 1.02)	
≥ 65	66.7 (39.1 – 86.2)	0.46 (0.1 – 2.04)	0.23
**Majority of patients comes from rural/urban area**			
Majority from rural area	73.4 (65 – 80.4)	1 (reference)	
Majority from urban or agglomeration area	61.9 (53.6 – 69.5)	0.53 (0.30 – 0.94)	
Equally urban or agglomeration/rural	75.6 (65.1 – 83.8)	0.83 (0.4 – 1.69)	0.079
**Self-rated information level**			
Rather bad	55.4 (44.1 – 66.2)	1 (reference)	
Middle	69.9 (62.7 – 76.3)	1.56 (0.86 – 2.87)	
Rather good	77.8 (68.2 – 85.1)	2.49 (1.22 – 5.1)	0.041
**Complementary medicine certificate**			
None	63.7 (58.0 – 69.1)	1 (reference)	
At least one^c^	96.6 (88.3 – 99.0)	16.1 (3.74 – 69.2)	<0.0001

Percentage of GPs who report at least one EMF consultation, stratified by GP-characteristics. ^a^Odds ratio of reporting at least one EMF consultation after multiple logistic regression (model variables: sex, age group, urban/rural patient collective, self rated information level and complementary-medicine certificate). ^b^p-value of likelihood ratio test after logistic regression. ^c^At least one of the following: traditional Chinese medicine/acupuncture, neural therapy, homeopathy or anthroposophical medicine

**Table 4:** Number of GPs who believed that EMFs can cause symptoms

**all GPs**	***% (95% C.I.)***	***OR (95% C.I.)^a^***	***p^b^***
	***61.4*** ***(56.1 – 66.4)***		
**Sex**			
Male	57.8 (51.9 – 63.5)	1 (reference)	
Female	76.1 (64.7 – 84.7)	1.76 (0.89 – 3.53)	0.095
**Age group**			
<35 – 44	70.3 (58.2 – 80.1)	1 (reference)	
45 – 54	66.7 (58.1 – 74.3)	0.76 (0.38 – 1.53)	
55 – 64	55.1 (46.7 – 63.1)	0.65 (0.33 – 1.29)	
≥ 65	25 (8.9 – 53.2)	0.22 (0.04 – 1.2)	0.25
**Majority of patients comes from rural/urban area**			
Majority from rural area	63.7 (55 – 71.6)	1 (reference)	
Majority from urban or agglomeration area	54.7 (46.4 – 62.7)	0.57 (0.33 – 0.99)	
Equally urban or agglomeration/rural	70.5 (59.6 – 79.5)	0.85 (0.43 – 1.68)	0.12
**Self-rated information level**			
Rather bad	58.1 (46.7 – 68.7)	1 (reference)	
Middle	66.5 (59.1 – 73.1)	1.22 (0.67 – 2.23)	
Rather good	56.7 (46.4 – 66.4)	0.76 (0.39 – 1.5)	0.27
**Complementary-medicine certificate**			
None	54.6 (48.8 – 60.3)	1 (reference)	
At least one^c^	94.8 (85.9 – 98.2)	13.75 (4.09 – 46.2)	<0.0001

Percentage of GPs who believe that EMF can cause symptoms, stratified by GP-characteristics. ^a^Odds ratio of believing that EMF can cause symptoms compared to not believing or being unsure about it after multiple logistic regression (model variables: sex, age group, urban/rural patient collective, self rated information level and complementary-medicine certificate). ^b^p-value after likelihood ratio test ^c^At least one of the following: traditional Chinese medicine/acupuncture, neural therapy, homeopathy or anthroposophical medicine.
